# Clinical Predictors and Recurrence Characteristics Following Radiotherapy for Primary Central Nervous System Lymphoma: A Retrospective Cohort Study

**DOI:** 10.3390/cancers17132176

**Published:** 2025-06-27

**Authors:** Jan Carl Bigge, Stephanie Bendrich, Hannes Treiber, Enver Aydilek, Nils Brökers, Gerald Georg Wulf, Carla Marie Zwerenz, Mahalia Zoe Anczykowski, Sandra Donath, Rami A. El Shafie, Lisa-Antonia von Diest, Jan Tobias Oelmann, Markus Anton Schirmer, Leif Hendrik Dröge, Martin Leu, Björn Chapuy, Stefan Rieken, Manuel Guhlich

**Affiliations:** 1 Clinic of Radiotherapy and Radiation Oncology, University Medical Center Göttingen, 37075 Göttingen, Germany; jancarl.bigge@stud.uni-goettingen.de (J.C.B.); stephanie.bendrich@med.uni-goettingen.de (S.B.); carlamarie.zwerenz@med.uni-goettingen.de (C.M.Z.); mahalia-zoe.anczykowski@med.uni-goettingen.de (M.Z.A.); sandra.donath@med.uni-goettingen.de (S.D.); rami.elshafie@med.uni-goettingen.de (R.A.E.S.); lisa-antonia.diest@med.uni-goettingen.de (L.-A.v.D.); jan.oelmann-avendano@med.uni-goettingen.de (J.T.O.); mschirmer@med.uni-goettingen.de (M.A.S.); hendrik.droege@med.uni-goettingen.de (L.H.D.); martin.leu@med.uni-goettingen.de (M.L.); stefan.rieken@med.uni-goettingen.de (S.R.); 2 Department of Hematology and Medical Oncology, University Medical Center Göttingen, 37075 Göttingen, Germanyenver.aydilek@med.uni-goettingen.de (E.A.); nils.broekers@med.uni-goettingen.de (N.B.); gerald.wulf@med.uni-goettingen.de (G.G.W.); bjoern.chapuy@charite.de (B.C.); 3 Department of Hematology, Oncology, and Cancer Immunology, Charité, Campus Benjamin Franklin, Charité-Universitätsmedizin Berlin, Corporate Member of Freie Universität Berlin and Humboldt-Universität zu Berlin, 12203 Berlin, Germany; 4 German Cancer Consortium, Partner Site Berlin, a Partnership Between German Cancer Consortium and Charité-Universitätsmedizin Berlin, 12203 Berlin, Germany

**Keywords:** primary central nervous system lymphoma, PCNSL, radiotherapy, overall survival, prognostic factors, real-world-analysis, seizures

## Abstract

**Background/Objectives**: Primary central nervous system lymphoma (PCNSL) is a rare but aggressive tumor, primarily affecting elderly patients. Radiotherapy (RT) remains an important treatment option, particularly for patients who are ineligible for systemic chemotherapy. This study aims to identify prognostic factors and evaluate recurrence patterns in a real-world cohort of PCNSL patients treated with RT. **Methods**: We retrospectively analyzed 64 PCNSL patients treated with radiotherapy at our institution between 2000 and 2022. Clinical characteristics, treatment details, and outcomes were collected by chart review. Overall survival (OS) was analyzed using Kaplan–Meier and Cox regression methods. Recurrence patterns were assessed based on available post-treatment imaging. **Results**: Median patient age was 71 years (range: 31–83); 53.1% had an Eastern Cooperative Oncology Group (ECOG) performance status ≥2. Radiotherapy was used as first-line treatment in 62.5% of cases, primarily due to contraindications to chemotherapy. Median OS was 10 months from diagnosis. Age, poor performance status, seizures at presentation, absence of systemic therapy, incomplete radiotherapy, and <80% applied dose of planned radiotherapy were associated with inferior OS in our univariable analysis. Multivariable analysis confirmed age, systemic therapy, seizures, and radiotherapy dose <80% as independent predictors. Among twenty-nine patients with imaging follow-up, eight recurrences after RT were documented: six of those within, and two outside of the initially affected areas. All recurrences occurred within previously irradiated areas. **Conclusions**: This study confirms known negative prognostic factors in PCNSL and underscores the importance of systemic chemotherapy for curatively intended treatments aiming for prolonged survival. The recurrence patterns observed question the added benefit of whole-brain irradiation in preventing distant relapses. These findings support the need for prospective trials to optimize radiotherapy strategies while balancing efficacy and neurotoxicity.

## Simple Summary

Primary central nervous system lymphoma is a rare but aggressive cancer that mostly affects older adults who often present with reduced performance status at first diagnosis. In this study, we reviewed the treatment courses and outcomes of 64 patients who received radiotherapy as part of their treatment. We aimed to understand which clinical factors influence patients’ survival and how radiotherapy can benefit patients who are not eligible for chemotherapy. Our analysis highlights that age ≥ 70 years, reduced performance status, seizures at diagnosis, and incomplete radiotherapy were linked to dismal outcomes. Interestingly, 80% of tumor relapses occurred within the initial tumor region, raising questions about the benefit of treating the entire brain. Our results provide insight into how radiotherapy could be better tailored to individual patients and highlight the need for further research to improve treatment strategies while minimizing side effects.

## 1. Introduction

Diffuse large B-cell lymphoma presenting as primary central nervous system (CNS) lymphoma (PCNSL) represents a rare form of Non-Hodgkin lymphoma, wherein brain, spinal cord, leptomeninges, and/or eyes are the only involved sites of disease. PCNSL accounts for approximately 4% of all primary malignant CNS tumors [1]. While the disease is confined to the CNS, it shares histopathological features with systemic diffuse large B-cell lymphoma, yet exhibits unique biological behavior and treatment responses due to the sanctuary nature of the CNS.

Radiotherapy, particularly whole-brain radiotherapy (WBRT), has historically played a pivotal role in the management of PCNSL, either as a primary modality for patients ineligible for chemotherapy or as consolidation following induction with high-dose systemic regimens [2,3,4]. However, its role has been re-evaluated over time due to concerns regarding late neurotoxicity and the emergence of more effective systemic treatment strategies [5,6,7].

PCNSL typically presents in older adults and is associated with aggressive clinical behavior, including rapidly progressive focal neurological deficits, neurocognitive impairment, and increased intracranial pressure. Without treatment, prognosis is dismal. The standard of care for fit patients consists of high-dose methotrexate (HD-MTX)-based chemotherapy, often in combination with rituximab and other agents such as cytarabine or thiotepa [2,6,8]. This approach has substantially improved outcomes, with reported median overall survival (OS) of up to 121 months in highly selected clinical trial cohorts [8]. However, a substantial proportion of patients—especially the elderly or frail—are ineligible for such regimens due to age-related vulnerability, comorbidities, or poor performance status. In addition, relapse and refractory disease remain critical therapeutic challenges, with limited evidence to guide second-line decision-making [9,10].

In this context, radiotherapy continues to have an important, albeit evolving, role. For chemotherapy-ineligible patients, WBRT remains a cornerstone of palliative and sometimes definitive treatment, offering rapid symptom relief and transient disease control [11]. For patients responding to systemic induction therapy, consolidative radiotherapy may help reduce CNS relapse rates, although its contribution to long-term neurotoxicity remains a matter of debate [8,12]. Particularly in older individuals, delayed cognitive deterioration after WBRT has prompted the evaluation of reduced-dose protocols and more focal radiotherapeutic strategies in prospective studies [12,13,14].

Despite advances in systemic therapy—including the implementation of high-dose chemotherapy with autologous stem cell transplantation, as well as novel agents such as ibrutinib, lenalidomide, and immune checkpoint inhibitors—radiotherapy remains a clinically relevant treatment option in selected patients [15,16,17]. Nonetheless, robust comparative data on the optimal timing, indication, and impact of radiotherapy in the real-world setting are scarce, especially outside of clinical trials.

In this retrospective study, we analyze the clinical course, treatment approaches, and outcomes of patients with PCNSL who received radiotherapy as part of their treatment course. By comparing patient characteristics and therapeutic responses across both first-line (primary treatment, *n* = 38; consolidation, *n* = 2) and second-line settings (progression during chemotherapy, *n* = 10; progression after chemotherapy, *n* = 14), we aim to provide real-world insights into the efficacy and tolerability of radiotherapy in this heterogeneous patient population. Our findings may help to refine treatment algorithms and identify subgroups of patients who derive particular benefit from the integration of radiotherapy into multimodal PCNSL therapy.

## 2. Materials and Methods

### 2.1. Patients and Study Design

This single-center study retrospectively analyzed patients receiving RT for PCNSL at our institution between 01/2000 and 12/2022. Patients and their respective diagnoses were identified by systematic keyword screening for “lymphoma” in tumor board protocols and manually pruned in the following step: Inclusion criteria required that patients had received at least one fraction of radiotherapy for cerebral lymphoma, either confirmed by histopathological diagnosis (*n* = 62; 97%) or, in the absence of histopathological verification, based on an interdisciplinary tumor board decision deeming the diagnosis highly probable and recommending treatment accordingly (*n* = 2; 3%). Data were extracted from physical patient records and RT treatment planning systems (Varian Eclipse, version 15.6, Varian Medical Systems, Palo Alto, CA, USA). Patient follow-up was evaluated through screening of hospital intern data processing systems (ixserv.4, version R20.3, ix.mid software technology, Köln, Germany) and ONKOSTAR (version 2.9.8, IT-Choice Software AG, Karlsruhe, Germany). The study was conducted in accordance with the Declaration of Helsinki and approved by the Ethics Committee of University Medical Center Goettingen (protocol code 17/11/21, date of approval: 25 November 2021).

### 2.2. Treatment Planning and Administration of Radiotherapy

Treatment consisted of whole-brain irradiation (whole brain radiotherapy, WBRT) with (n = 7, 10.9%) or without (n = 57, 89.1%) tumor boost. WBRT was delivered via a standard helmet field technique (HF), including cervical vertebrae 1 and 2, employing bilateral opposed lateral fields in 95.3% of cases (n = 61); 3 patients received intensity-modulated radiotherapy (IMRT) or volumetric modulated arc therapy (VMAT) (4.7%, Table 1). Target delineation was applied according to hospital internal standard treatment protocols varying over time: Margins from clinical target volume (CTV) to planning target volume (PTV) differed between 5–10 mm; in the case of tumor boost, gross tumor volume (GTV) to CTV-Margin was 10 mm and CTV to PTV was 5 mm. Median prescribed dose including tumor boost was 45 Gy in 1.8 Gy/fraction (*n* = 45, 70.3%) and ranged from 30 Gy (*n* = 6, 3 Gy per fraction, due to poor general condition) up to 59,4 Gy (*n* = 2, according to the RTOG 88-06 trial, [3]).

### 2.3. Statistical Analysis

Statistical analyses were performed using SPSS (v26) and R (v4.0.2) with the “KMWin” (Kaplan–Meier for Windows) plugin [18]. Survival data were displayed by Kaplan–Meier plots; survival time comparisons were performed by log-rank tests. Univariable Cox regression was applied to assess the impact of variables on survival. We considered *p*-values < 0.05 as statistically significant. Univariably significant variables were consecutively tested in a multivariable fashion.

## 3. Results

### 3.1. Patient, Disease and Radiotherapy Treatment Characteristics

A total of 64 patients were included in this analysis. The median age was 71 years, ranging from 31 to 83 years, and female patients comprised the majority of the cohort (42 patients, 65.6%). At the start of radiotherapy, 30 patients (46.9%) had an ECOG performance status of 0 or 1, whereas 34 patients (53.1%) had a status of 2 or higher. Two out of sixty-four patients (3%) did not have histopathological confirmation (Section 2.1). Radiotherapy was administered as first-line treatment in 40 patients (62.5%) and as second-line treatment in 24 patients (37.5%). Treatment was discontinued prematurely in 27 patients (42.2%). The median prescribed radiotherapy dose was 45.0 Gy, with a range of 31.8 to 59.4 Gy. A total of 11 patients (17.2%) required concomitant anticonvulsant medication during radiotherapy. Please refer to Table 1 for details concerning patient, disease, and RT treatment characteristics.

### 3.2. Survival Parameters and Potential Influencers

The median OS from initial diagnosis for the entire cohort was 10 months (95% CI: 2.9–17). Among patients who received RT as part of first-line treatment—excluding those two treated with consolidative intent—the median OS was 6 months (95% CI: 2.8–9.1). In contrast, patients who underwent RT in the second-line setting had a median OS of 34 months (95% CI: 21.3–46.6; *p* = 0.003 vs. first-line treatment, log-rank test). Of the 2 patients who received consolidative treatment, 1 was still alive at the 127-month follow-up, while the other was lost to follow-up due to relocation to another country.

**Figure 1:**
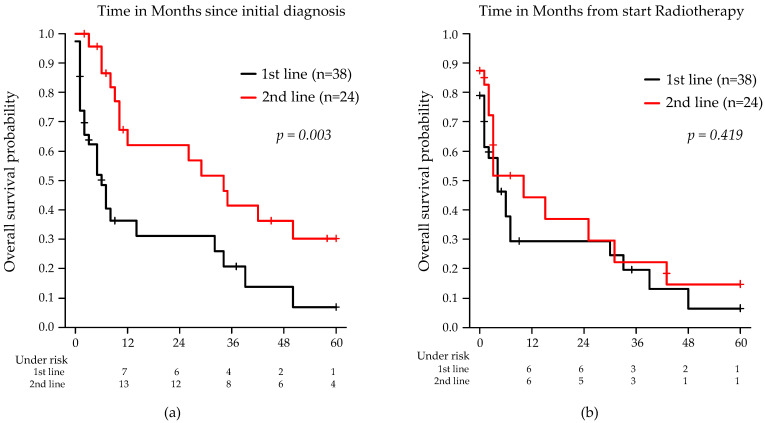
Kaplan–Meier estimates of OS stratified by RT in first- or second-line treatment of PCNSL: (**a**) from initial diagnosis; (**b**) from the start of RT. Note: Two patients who received consolidative RT were excluded from the analysis.

**Figure 2:**
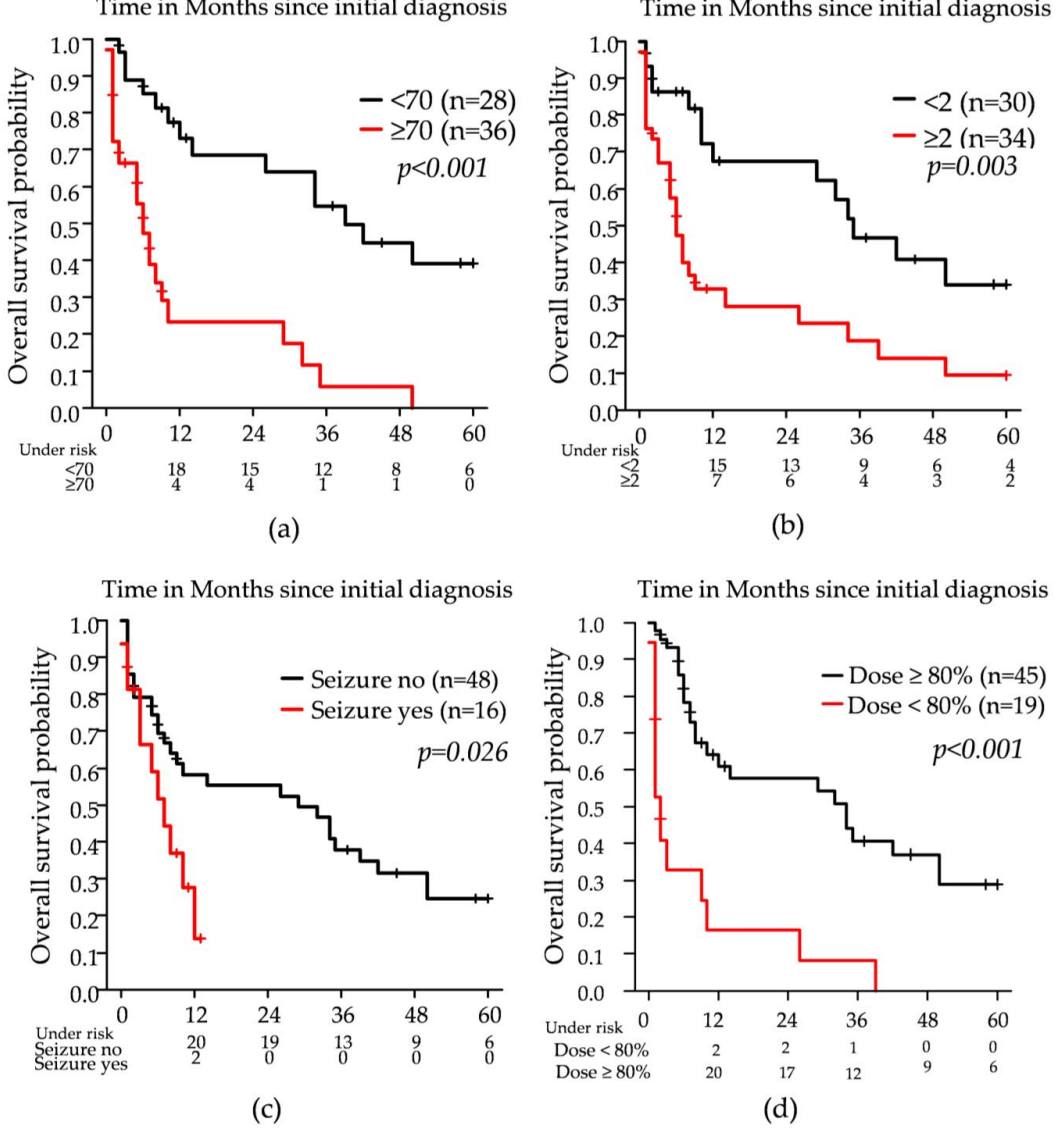
Kaplan–Meier estimates of overall survival (OS) stratified by clinical prognostic factors: (**a**) age ≥ 70 vs. <70 years; (**b**) baseline ECOG performance status <2 vs. ≥2; (**c**) presence vs. absence of seizures at diagnosis; (**d**) radiotherapy dose application (<80% vs. ≥80%).

When OS was calculated from the initiation of RT, the median OS was 4 months (95% CI: 0.5–7.5) for first-line RT patients and 10 months (95% CI: 3.4–16.6) for those receiving RT in the second-line setting (*p* = 0.42, log-rank test). Kaplan–Meier survival curves illustrating the survival outcomes are shown in Figure 1 and Figure 2.

Older age (≥70 years), baseline ECOG ≥ 2, systemic therapy, seizures as a presenting symptom, RT completion as intended, receipt of ≥80% of the prescribed RT dose, and RT line (first vs. second) were all associated with OS in univariable Cox regression analysis. In the multivariable model, age, systemic therapy, seizures, and administration of ≥80% of the RT dose remained statistically significant. Detailed results are provided in Table 2.

Radiotherapy dose completion was assessed relative to the initially prescribed total dose. Patients receiving <80% of the intended dose (n = 19) had experienced early discontinuation of treatment, predominantly due to clinical deterioration. In this subgroup, the applied doses ranged from 5.4 Gy to 34.2 Gy, corresponding to 12% to 76% of the planned regimen. Most patients in this group received between 20% and 60% of the intended dose.

The median delivered dose was 21.6 Gy (interquartile range (IQR) 13.2–27.9 Gy). The 80% threshold was pragmatically chosen to differentiate between patients with marked treatment deviations and those who completed radiotherapy as planned, thus enabling stratified survival analysis based on treatment adherence.

### 3.3. Local Recurrences

**Figure 3:**
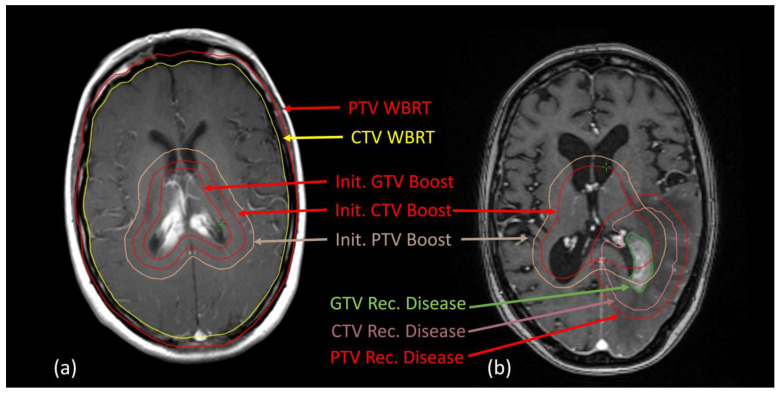
Representative MRI slices matched with RT planning CT. (**a**) Initial lymphoma manifestation in the area of mid/posterior corpus callosum and corresponding target volumes, (**b**) local recurrence at the posterior horn of the left lateral ventricle partly overlapping the initial boost volume, 36 months after initial RT treatment. Abbreviations: GTV = gross tumor volume, CTV = clinical target volume, PTV = planning target volume. Treatment was applied via volumetric modulated arc therapy, initial dosing: PTV whole-brain RT in HF volume: 23.4 Gy/1.8 Gy/fraction; PTV Boost: 21.6 Gy/1.8 Gy; PTV recurrent disease: 36 Gy/1.8 Gy/fraction.

Of the total cohort, 45.3% (29/64 patients) underwent post-radiotherapy cerebral imaging, including six patients with contrast-enhanced cerebral CT and twenty-three with contrast-enhanced MRI. Intracerebral recurrence was identified in eight patients—seven by MRI and one by CT. Six out of these eight patients showed local recurrence within or overlapping the original lymphoma manifestation; four of those had initial HF treatment to 45 Gy, two had HF and sequential local boost (37.5 Gy WBRT + 7.5 Gy Boost; 23.4 Gy WBRT + 21.6 Gy Boost). Two out of eight intracerebral recurrences were distant to the initial manifestation; both patients had previously received WBRT in HF technique (45 Gy, 54 Gy), therefore including the area of the following recurrence. Reirradiation was applied in two patients: one with HF technique up to 19.8 Gy (initially: 45 Gy), and one with local reirradiation up to 36 Gy (initial RT 23.4/21.6 Gy). Please refer to Figure 3 for an example of a local recurrence treated with focal re-irradiation.

## 4. Discussion

In this retrospective analysis of 64 patients diagnosed with PCNSL who received RT as part of their treatment regimen, several clinically relevant observations were made. The median age of 71 years indicated that the cohort is a representative PCNSL cohort aligning with the known epidemiology of PCNSL, which predominantly affects elderly individuals. More than half of the patients (53.1%) presented with an ECOG performance status ≥2 at the start of RT, indicating a clinically highly vulnerable population. Seizures were the initial symptom in 25% of patients—an indicator of potentially advanced disease. Furthermore, in 27 cases, comprising 43% of the whole cohort, RT was aborted prematurely, mainly due to a further deterioration in general condition.

RT was administered as first-line therapy in the majority of patients (62.5%), mainly due to insufficient general condition, contraindication for chemotherapy regimens, or toxicity-induced therapy discontinuation (37/40 patients, 92.5%). This reflects both institutional standards—reserving high-dose chemotherapy and autologous stem cell transplantation for fit patients—and the prognostically adverse composition of our cohort. A limitation of our study is the absence of histological confirmation in two patients (3%). While this reflects real-world challenges in frail patients with typical imaging and clinical presentation, it may slightly affect diagnostic accuracy and the interpretation of outcomes.

Median OS from diagnosis was 10 months and was significantly longer in the second-line group. However, when using RT initiation as the starting point, no statistically significant OS difference remained between first- and second-line RT, underlining the central role of systemic chemotherapy. In univariable analysis, age ≥ 70, ECOG ≥ 2, seizure presentation, absence of systemic therapy, incomplete RT, and dose < 80% were all associated with inferior OS. Multivariable analysis confirmed age, systemic therapy, seizures, and RT dose ≥80% as independent predictors. These results corroborate existing data showing that advanced age and poor performance status are among the strongest negative predictors for survival in PCNSL—primarily by representing a contraindication for systemic therapy [10,15,16,19]. If RT is applied as the only therapeutic option, the completion and dose intensity of RT might appear to be critical determinants of treatment effectiveness. However, in our cohort, these results most likely reflect (further) deterioration of general condition during treatment due to PCNSL and, subsequently, very limited short-term survival. Thus, in very frail patients, the decision to initiate RT must weigh potential benefit against limited prognosis. However, it should be noted that WBRT has shown superiority to best supportive care in terms of survival [11].

Interestingly, seizure presentation emerged as an independent predictor of poor outcome. Although seizures are not commonly discussed as a prognostic marker in PCNSL, they may reflect underlying extensive cortical involvement or elevated intracranial pressure, which could contribute to worse clinical status and reduced treatment tolerance [17].

Consolidative RT was remarkably low (n = 2, 3.1%), indicating relevant restraints due to concerns of negative neurocognitive outcomes after combined treatment [20].

It is known that a multitude of factors can influence neurocognitive outcome: higher age, previous systemic therapy, as well as high RT dose seem to be of highest relevance [21]. In recent years, encouraging prospective data indicating suitability of low-dose (ld-) WBRT (23.4 Gy/1.8 Gy/fr) following systemic therapy without an increase in neurotoxicity and improved PFS have been reported [22]. The use of ld-WBRT, supported by large-scale retrospective data, have since been widely implemented in various guidelines and are now part of routine treatment at our institution [23,24,25].

An ongoing debate among radiation oncology experts concerns the use of WBRT versus partial-brain radiotherapy (PBRT) [14,26,27]. We assessed recurrence patterns in 29 patients with follow-up imaging (45% of the cohort, 78% of those completing therapy). Consistent with literature indicating that only 20% of recurrences are detected via routine imaging, eight intracerebral recurrences were identified [28,29]. Notably, both patients with distant recurrences had previously received WBRT with total doses of 45 Gy and 54 Gy, respectively. This finding aligns with the pattern observed in the six patients who relapsed locally, all of whom had also received radiotherapy to a dose of 45 Gy, with or without an additional boost. Notably, all recurrences—whether local or distant—occurred within areas that had received doses of 45 Gy or higher. Similar findings have been reported in retrospective data from Japan, where both in-field and out-of-field recurrences were documented [30]. Data from Italy indicated that up to 60% of relapses occurred outside the initially irradiated volumes [12]. These discrepancies underline the heterogeneity of recurrence patterns and the challenges in defining optimal target volumes. Recent radiotherapy approaches such as hippocampal-sparing whole-brain irradiation aim to preserve cognitive function without compromising oncological outcomes, particularly in the context of primary or consolidative treatment. However, the feasibility of such techniques in PCNSL remains uncertain due to the frequent periventricular location of lesions and the currently limited clinical evidence supporting their use in this specific setting [12,31].

Currently, recommendations concerning target volume delineation and the use of (simultaneously integrated) boost concepts differ between guidelines [24,32,33]. Our findings—while based on a retrospective design and a limited patient cohort—may question the assumption that WBRT offers superior protection against distant intracranial recurrence. Despite extended target volumes and relatively high total doses, WBRT did not prevent relapses outside the initially involved sites. However, as only a small number of patients (n = 7) received a focal radiotherapy boost, the observed recurrence pattern must be interpreted with caution. No definitive conclusions can be drawn regarding the comparative benefit of whole-brain versus focal irradiation. Additionally, the occurrence of relapses within previously irradiated areas may suggest radioresistance rather than insufficient spatial coverage. Still, a potential benefit of WBRT in preventing future, currently subclinical relapses cannot be excluded, particularly given the retrospective nature of our analysis. For example, data from a French survey of 79 patients reported of significantly shorter OS when PBRT was applied as opposed to WBRT [34]. Reducing total WBRT dose appears to be a viable option not only in the case of consolidation [26]. Contrarily, there are also emerging data on stereotactic ablative RT specifically targeting CNS lesions or combinations of ld-WBRT with SBRT-boost [13,35,36].

In conclusion, both the RT field size (WBRT versus focal) and dose strategies, including ld-WBRT with or without boost, can significantly impact neurocognitive outcomes but remain insufficiently defined by current evidence. Our real-world data contribute to the existing literature by reaffirming established prognostic factors and questioning the assumed oncological superiority of whole-brain radiotherapy. These findings highlight the urgent need for prospective clinical trials to refine radiotherapy approaches and improve personalized treatment for patients with PCNSL.

## 5. Conclusions

This retrospective analysis of PCNSL patients receiving RT highlights the clinical relevance of known prognostic factors such as age and the ability to receive systemic chemotherapy. Importantly, seizure presentation and incomplete RT—both indicative of aggressive disease and limited treatment tolerance—were also associated with significantly inferior outcomes.

All observed relapses, including distant ones, occurred within previously irradiated regions, suggesting dose resistance rather than insufficient coverage. Given the growing emphasis on minimizing neurotoxicity, approaches such as low-dose WBRT and focal boost concepts warrant further investigation. Overall, our real-world data underscore the urgent need for prospective trials to refine radiotherapy strategies in PCNSL and guide individualized treatment decisions based on clinical and biological risk profiles.

## Figures and Tables

**Table 1:** Patient, disease and treatment characteristics. ECOG = Eastern Cooperative Oncology Group; RT = Radiotherapy; CTx = Chemotherapy (any); 3D-cRT = 3dimensional conformal Radiotherapy; IMRT/VMAT = Intensity-modulated Radiotherapy/Volumetric modulated Arc Therapy; * Applicable for n = 39 Pat.

Patients, N (%)	64 (100%)
Age (years), median (min–max)	71 (31–83)
Sex: female:male, N (%)	42 (65.6):22 (34.4)
Body Mass Index, median (min-max)	26.3 (15.4–35)
ECOG Status at Initiation of Radiotherapy: N (%)	
0	5 (7.8)
1	25 (39.1)
2	18 (28.1)
3	11 (17.2)
4	5 (7.8)
Initial disease characteristics N (%)	
Ki67% ≥ 90% *	6 (9.4)
Solitary Lesion	34 (53.1)
Initial Seizure	16 (25)
Histopathologically confirmed disease	62 (96.9)
Indication for Radiotherapy	
First line RT	40 (62.5)
Unfit for CTx	32 (50)
CTx refused by patient	1 (1.6)
CTx aborted due to toxicity	5 (7.8)
Consolidating RT	2 (3.1)
Second line RT	24 (37.5)
Progressive Disease post CTx	14 (21.9)
Progressive Disease during CTx	10 (15.6)
Radiotherapy Treatment Characteristics	
RT completed as intended	37 (57.8)
RT aborted prematurely	27 (42.2)
≥80% of intended RT dose applied	45 (70.3)
Glucocorticoids during RT	51 (79.7)
Anticonvulsiva during RT	11 (17.2)
Dose, median (min–max)	45.0 Gy (31.8–59.4)
3DcRT	61 (95.3)
IMRT/VMAT	3 (4.7)

**Table 2:** Influence of potential prognostic factors on patients’ OS from initial diagnosis. Calculations were done by Cox regression analyses. *p* values < 0.05 were considered statistically significant and are depicted in bold. Variables with *p* < 0.1 in univariable analysis were consecutively tested in a multivariable Cox regression model. CI = confidence interval; CCI = Charlson Comorbidity Index, dichotomized by median (=5), Gy = Gray, n.s. = not significant; ECOG = Eastern Cooperative Oncology Group; RT = Radiotherapy * only applicable for *n* = 17 patients due to missing data.

Variable	Hazard Ratio (95% CI) Univariable	*p*-Value	Hazard Ratio (95% CI) Multivariable	*p*-Value
Age at diagnosis (years) ≥70 (*n* = 36) vs. <70 (*n* = 28)	4.30 (2.13–8.66)	**<0.001**	5.02 (2.23–11.29)	**<0.001**
Female (*n* = 42) vs. Male (*n* = 22)	0.87(0.45–1.57)	0.585		
Initial ECOG: ≥2 (n = 34) vs. <2 (n = 30)	2.48 (1.30–4.74)	**0.006**		n.s.
CCI: <5 (n = 30) vs. ≥6 (n = 34)	1.76 (0.93–3.32)	0.082		
Smoker (n = 12) vs. non-Smoker (n = 35)	0.923 (0.43–1.97)	0.725		
Systemic therapy: none (n = 31) vs. any (n = 33)	5.027 (2.47–10.20)	**<0.001**	2.69 (1.29–5.64)	**0.008**
Cerebral Lesions: multiple (n = 26) vs. solitary (n = 34)	1.07 (0.56–2.05)	0.828		
KI 67 initially: ≥90 (n = 6) vs. <90 (n = 11) *	2.12 (0.76–5.90)	0.150		
Seizure (n = 16) vs. no seizure (n = 48)	2.240 (1.05–4.76)	**0.036**	3.67 (1.68–8.05)	**0.001**
RT: incomplete (n = 27) vs. completed as intended (n = 37)	3.89 (2.01–7.54)	**<0.001**		n.s.
RT dose applied as intended: ≥80% (n = 45) vs. <80% (n = 19)	0.21 (0.11–0.43)	**<0.001**	0.20 (0.09–0.43)	**<0.001**
Anticonvulsants during RT: yes (n = 11) vs. no (n = 45)	1.48 (0.71–3.10)	0.298		
Corticosteroids during RT: yes (n = 51) vs. no (n = 13)	1.55 (0.69–3.49)	0.294		
Dynamic RT (n = 3) vs. 3DcRT (n = 61)	0.32 (0.04–2.35)	0.263		
Boost: yes (n = 7) vs. no (n = 57)	0.67 (0.20–2.18)	0.50		
RT: Second-line (n = 24) vs. First-line (n = 38)	0.39 (0.20–0.76)	**0.006**		n.s.

## Data Availability

The datasets generated and/or analyzed in the current study are available from the corresponding author upon reasonable request.

## Abbreviations

The following abbreviations are used in this manuscript:

CCI	Charlson Comorbidity Index
CI	confidence interval
CTV	clinical target volume
CTx	Chemotherapy
ECOG	Eastern Cooperative Oncology Group
GTV	gross tumor volume
Gy	Gray
HF	helmet field technique
IMRT	intensity-modulated radiotherapy
ld	Low-dose
n.s.	not significant
OS	Overall survival
PCNSL	Primary central nervous system lymphoma
PTV	planning target volume
RT	Radiotherapy
VMAT	volumetric modulated arc therapy
WBRT	whole-brain radiotherapy
3D-cRT	3dimensional conformal Radiotherapy
